# Intra-articular hyaluronic acid injections for hip osteoarthritis: a level I systematic review

**DOI:** 10.1007/s00590-025-04292-7

**Published:** 2025-05-09

**Authors:** Filippo Migliorini, Marco Pilone, Manuel Giovanni Mazzoleni, Luise Schäfer, Dragana Katusic, Nicola Maffulli

**Affiliations:** 1 Department of Trauma and Reconstructive Surgery, University Hospital in Halle, Martin-Luther University Halle-Wittenberg, Halle, Germany; 2 Department of Life Sciences, Health, and Health Professions, Link Campus University, Rome, Italy; 3 University of Milan, Milan, Italy; 4 Department of Orthopaedic and Trauma Surgery, Academic Hospital of Bolzano, Bolzano, Italy; 5 Department of Orthopaedic and Trauma Surgery, Eifelklinik St.Brigida, Simmerath, Germany; 6 Faculty of Medicine and Psychology, Sapienza University of Rome, Rome, Italy; 7 Centre for Sports and Exercise Medicine, Queen Mary University of London, London, UK; 8 School of Pharmacy and Bioengineering, Keele University Faculty of Medicine, Stoke on Trent, United Kingdom

**Keywords:** Hip, Osteoarthritis, Hyaluronic acid, Injections

## Abstract

**Purpose:**

The present systematic review investigated the efficacy of intra-articular hyaluronic acid (HA) viscosupplementation for hip osteoarthritis (OA) in patient-reported outcome measures (PROMs) and whether different molecular weights of HA are associated with different outcomes.

**Methods:**

This study was conducted according to the 2020 PRISMA statement. In January 2025, PubMed, Web of Science, Google Scholar, and Embase were accessed. All the randomised controlled trials (RCTs) evaluating the efficacy of intra-articular HA injections in the hip for OA were included.

**Results:**

Nine hundred and eighty-two patients (56% women, mean age 62.2 ± 4.0 years, mean follow-up 6.4 ± 2.7 months, mean BMI 27.5 ± 2.1 kg/m^2^) were analysed. Patients receiving high molecular weight (HMW) and low molecular weight (LMW) HA showed significant improvements in Western Ontario and McMaster Universities Osteoarthritis Index (WOMAC) and visual analogue scale (VAS) scores (*P* < 0.05). No significant differences in VAS or WOMAC were observed among groups at 3–4 months of follow-up. However, at 4–6 months, the HMW HA group exhibited significantly lower VAS scores compared to the medium molecular weight (MMW) (mean difference, MD − 1.4, 95% CI − 2.1 to − 0.7, *P *< 0.0001), placebo (MD − 1.6, 95% CI − 2.1 to − 1.1, *P* < 0.0001), and control (MD − 1.3, 95% CI − 1.8 to − 0.8, *P *< 0.0001) groups. WOMAC scores at 4–6 months demonstrated that both HMW and MMW HA performed better than the control group (*P* < 0.0001), but no significant difference was observed between HMW and MMW (*P* = 1.0).

**Conclusion:**

Intra-articular injections of HA effectively reduce knee OA symptoms. Moreover, HMW HA performs better than MMW HA at a mean of 4–6 months of follow-up.

**Level of evidence:**

Level I, systematic review of RCTs.

**Supplementary Information:**

The online version contains supplementary material available at 10.1007/s00590-025-04292-7.

## Introduction

Hip osteoarthritis (OA) is a progressive degenerative disease affecting the articular cartilage and surrounding hip tissues, resulting in diminished quality of life as a result of pain and functional limitations [1–4]. Globally, hip OA ranks among the leading causes of chronic disability, with approximately one in ten adults diagnosed clinically and nearly one in three demonstrating radiographic evidence of cartilage degeneration in industrialised countries [5–8]. Despite the success of total hip arthroplasty in managing end-stage hip OA [9–11], strategies to halt or slow its progression remain elusive, with current approaches primarily focussed on symptom management, including physiotherapy, oral non-steroidal anti-inflammatory drugs (NSAIDs), and intra-articular injections [12, 13].

The National Institute for Health and Care Excellence (NICE) guidelines advocate nonpharmacological and pharmacological management in early OA stages [14]. In this context, intra-articular injections, preferably guided by ultrasound for better accuracy, are considered only when adjunct to therapeutic exercise protocols [15, 16]. Different drugs have been studied for intra-articular administration, offering minimal systemic side effects compared to oral medications, with corticosteroids (CCs) being the most widely used [17, 18]. However, NICE guidelines caution against CCs injections given the inconsistent benefits on quality of life and function, alongside providing only short-term pain relief, with some studies suggesting a potential risk of rapidly progressive osteoarthritis [14, 19, 20].

Consequently, research has explored alternative injectable therapies for hip OA, including viscosupplementation [21–24]. Within OA joints, the chronic inflammatory environment impairs the turnover of hyaluronic acid (HA), a high-molecular-weight glycosaminoglycan polysaccharide abundant in normal human synovial fluid, which serves as a crucial biological lubricant [25, 26]. The reduction in the molecular weight and concentration of HA after the onset of OA impairs the lubricating and protective effect of the synovial fluid [27, 28]. Given the high viscosity at low shear rates and increased elasticity during rapid movements, HA viscosupplementation theoretically restores synovial fluid rheologic properties, thereby reducing joint friction and resistance and potentially preventing further cartilage degradation [29, 30]. The efficacy of injectable HA products varies within molecular weight (MW), with higher MW positively correlated with improved rheologic properties and residence time [31–33]. Additionally, intra-articular HA supplementation may exert biological cellular modifying effects, including anti-oxidative, anti-inflammatory, and analgesic actions [34, 35]. Clinical evidence suggests HA viscosupplementation is safe and effective in selected patients with OA, providing satisfactory pain relief and functional improvements [36–40]. However, fewer studies support the efficacy of HA in hip OA compared to knee OA, with existing literature exhibiting high study heterogeneity, low evidence levels, and considerable bias risks [41–43]. Despite its potential benefits, HA injection for hip OA is not widely recommended in international guidelines [16]. The NICE Guidelines discourage its use in UK clinical practice, given the possible risks of harm [14]. Moreover, HA injections are expensive, synthetically manufactured, and exhibit inconsistent effects on inflammation [43, 44].

Another intervention gaining popularity is platelet-rich plasma (PRP) intra-articular injection [45–47]. PRP is an autologous product derived from whole blood that contains elevated platelet levels and higher concentrations of growth factors [48, 49]. A recent meta-analysis suggests PRP injection as an alternative treatment option for hip OA with low—and moderate-quality evidence in pain reduction and functional improvement, but clear recommendations are lacking [50].

Therefore, the primary endpoint of the present systematic review aims to evaluate the efficacy of intra-articular HA viscosupplementation as a therapeutic intervention for hip OA, focussing on the highest level of evidence available. The secondary endpoint of the present study is to evaluate whether different molecular weights of HA are associated with different outcomes. Given the crucial role of HA in synovial fluid, we hypothesise that intra-articular viscosupplementation is an effective therapeutic intervention for hip OA. We expect that intra-articular HA injection, compared to placebo and PRP, will lead to improvements in patient-reported outcome measures (PROMS), as assessed by visual analogue scale (VAS) and Western Ontario McMaster Osteoarthritis Index (WOMAC) scores.

## Methods

### Eligibility criteria

All the randomised controlled trials (RCTs) investigating the efficacy of intra-articular HA injections in the hip for OA were accessed. Only studies published in peer-reviewed journals were considered. According to the author´s language capabilities, English, German, Italian, French and Spanish articles were eligible. Only studies with levels I of evidence, according to the Oxford Centre of Evidence-Based Medicine [51], were considered. Only studies which compared HA with other biologically active non-HA treatments (e.g. platelet-rich plasma or mesenchymal stem cells) were considered. Studies which evaluated intra-articular HA injections augmented with other biologically active compounds were not considered. Studies that were regarded as comparators of other non-infiltrative therapies were not eligible.

### Search strategy

This study was conducted according to the Preferred Reporting Items for Systematic Reviews and Meta-Analyses: the 2020 PRISMA statement [52]. The PICOTD algorithm was preliminarily established:

- P (Problem): hip OA;
- I (Intervention): intra-articular HA injections;
- C (Comparison): molecular weights of HA and control groups;
- O (Outcomes): PROMs, VAS and WOMAC.
- T (Timing): minimum of 3 months follow-up;
- D (Design): RCTs.

In January 2025, the following databases were accessed: PubMed, Web of Science, and Embase. No time constraint was set for the search. The Medical Subject Headings (MeSH) used for the database search are reported in the Appendix. No additional filters were used in the database search.

### Selection and data collection

Two authors (**&**) independently performed the database search. All the resulting titles were screened by hand, and the abstract was accessed if suitable. The full text of the abstracts which matched the topic was accessed. If the full text was not accessible or available, the article was not considered for inclusion. A cross reference of the bibliography of the full-text articles was also performed for inclusion. Disagreements were debated and mutually solved by the authors. In further disagreements, a third senior author (**) made the final decision.

### Data items

Two authors (**&**) independently performed data extraction. The following data at baseline were extracted: author, year of publication and journal, length of the follow-up, number of patients with related mean age and BMI. Data concerning the following PROMs were collected at baseline and the last follow-up: VAS and WOMAC [53]. Data were extracted in Microsoft Office Excel version 16.72 (Microsoft Corporation, Redmond, USA). Concerning the WOMAC score, 24 health-specific items covering pain (five items), stiffness (two items) and function (17 items) were assessed. The subscale scores for pain, stiffness and function are summed to produce the total score. Scores range from 0 (least pain) to 20 (highest pain) for pain, 0 (least stiffness) to 8 (greatest stiffness) for stiffness, 0 (best function) to 68 (worst function) for function and 0 (best health) to 96 (worst health) for the total score. The primary endpoint of the present study was to evaluate the efficacy of intra-articular HA injections. The efficacy was assessed as improving PROMs from baseline to the various follow-ups. The secondary endpoint of the present study was to evaluate whether different molecular weights of HA are associated with different outcomes and to compare their efficacy with a placebo and a control group. Placebo was considered any intra-articular injection performed with isotonic saline solution or anaesthetic. Control was considered any intra-articular injection performed with any biologically active compound (platelet-rich plasma, CSs). Low molecular weight (LMW) is defined as 500–1500 kDa, medium molecular weight (MMW) from 1500 to 3000 kDa, high molecular weight (HMW) from 3000 to 6000 kDa, and ultra-high molecular weight (UHMW) more than 6000 kDa [32, 54, 55].

### Methodological quality assessment and quality of the recommendations

The risk of bias was evaluated following the guidelines in the Cochrane Handbook for Systematic Reviews of Interventions [56]. Two reviewers (**&**) independently assessed the risk of bias in the extracted studies. Disagreements were solved by a third senior author (**). RCTs were evaluated using the revised Risk of Bias assessment tool (RoB2) [57, 58] of the Cochrane tool for assessing the Risk of Bias in randomised trials (RoB). The following endpoints were evaluated: bias arising from the randomisation process, bias based on the deviations from intended interventions, bias because of missing outcome data, bias in the measurement of the outcome, and bias in the selection of the reported result.

### Synthesis methods

The main author (**) performed the statistical analyses following the recommendations of the Cochrane Handbook for Systematic Reviews of Interventions [59]. The IBM SPSS software version 25 (International Business Machines Corporation, Armonk, USA) was used. Mean and standard deviation were used for descriptive statistics. The mean difference (MD) was calculated with a 95% confidence interval (CI) to evaluate the improvement from the baseline to the last follow-up. The paired *t* test was performed with values of *P* < 0.05 considered statistically significant. The analysis of variance (ANOVA) was used to assess baseline comparability and compare multiple continuous variables. For baseline comparability, values of *P* > 0.1 were considered satisfactory. For group comparisons, the CI was set at 95% in all the comparisons. Final values of *P* < 0.05 were considered statistically significant.

## Results

### Study selection

**Fig. 1:**
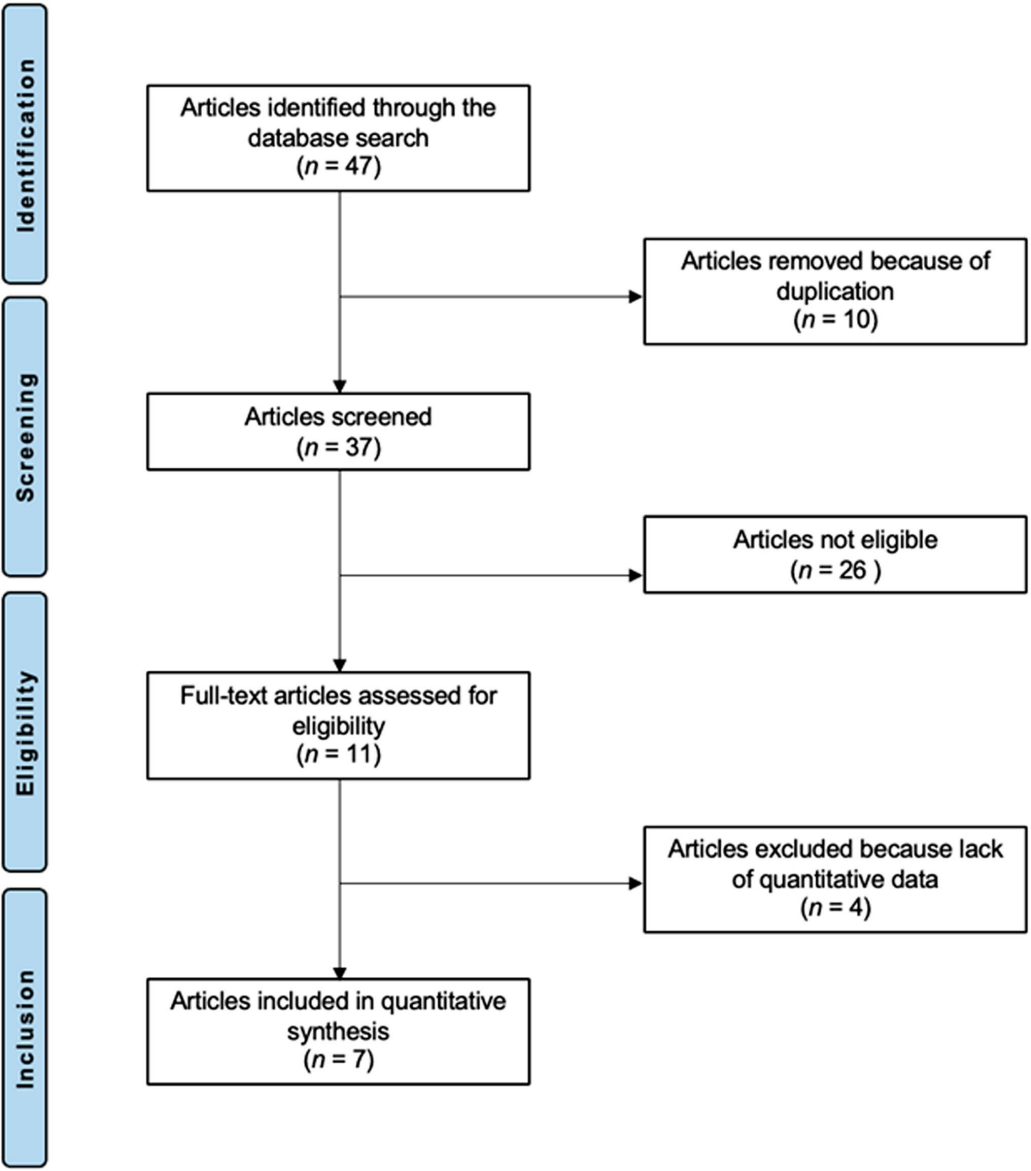
PRISMA flow chart of the literature search

The literature search resulted in 47 articles. Of these, 10 were excluded as they were duplicates. The remaining 37 articles were screened for eligibility. Of them, 26 articles were excluded as they did not match the eligibility criteria: study type and design (*N *= 13), language limitations (*N *= 4), and minimum follow-up shorter than 3 months (*N *= 8). Four more studies were excluded as they missed quantitative data under the outcomes of interest. Finally, seven RCTs were included in the present investigation. The results of the literature search are shown in Fig. 1.

### Methodological quality assessment

**Fig. 2:**
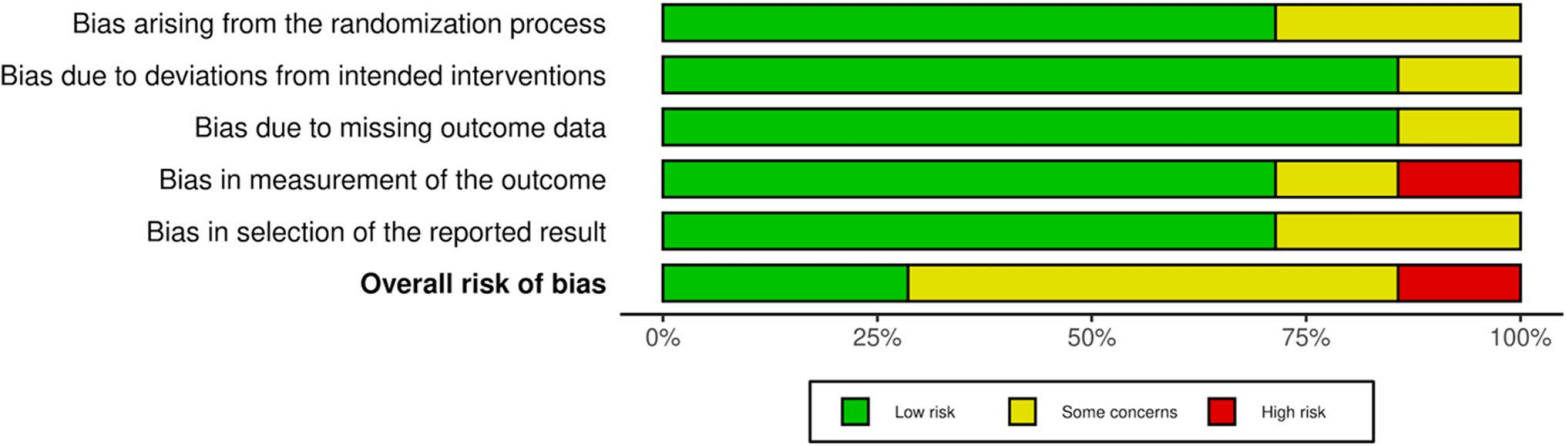
Cochrane risk of bias tool (RoB2)

The revised Cochrane risk of bias assessment tool (RoB2) was performed to investigate the risk of bias in all investigations included in the present review, as they were all RCTs. The assessment identified some concerns during the randomisation process. However, given the established comparability of the groups studied at baseline, bias arising from the randomisation process was rated with predominantly moderate risk. Risk of bias based on the deviations from the intended intervention, missing outcome data, the selection of the reported outcome, and the measurement of the outcome were occasionally noted with some concerns, leading to a low to moderate overall risk of bias in these domains. Missed assessor blinding resulted in a high risk of bias in measuring the outcome in one of the articles; in all other studies, a low to moderate risk was raised for this domain. Concluding, the risk of bias graph evidenced a good quality of the methodological assessment of RCTs (Fig. 2).

### Study characteristics and results of individual studies

Data from 982 patients were collected. The mean length of the last follow-up was 6.4 ± 2.7 months. 56% (505 of 902) patients were women. The mean age of the patients was 62.2 ± 4.0 years, and their mean BMI was 27.5 ± 2.1 kg/m^2^. Generalities and demographics of the included studies are shown in Table 1.

**Table 1:** Generalities and demographics of the included studies

Author, year	Journal	Last FU (months)	Group	Patients (*n*)	Mean age (year)	Female (%)	Mean BMI (kg/m^2^)
Brander et al. [60]	*Osteoarthritis Cartilage*	6	HMW	182		24	30.9
			Placebo	175		30	29.1
Doria et al. [61]	*Joints*	12	MMW	40	68.0		25.0
			Control (PRP)	40	67.3		24.3
Migliore et al. [62]	*Arthritis Res Ther*	6	MMW	22	68.0	45	25.6
			Placebo	20	67.0	50	24.8
Nouri et al. [63]	*BMC Musculoskelet Disord*	6	MMW	35	60.9	76	27.6
			Control (PRP)	35	58.2	69	27.7
Richette et al. [64]	*Arthritis Rheum*	3	LMW	42	60.8	64	26.7
			Placebo	43	59.5	53	26.4
Spritzer et al. [65]	*Phys Sportsmed*	6	HMW	150	59.0	52	29.3
			Control (CCs)	155	59.0	51	29.4
Tikiz et al. [66]	*Clin Rheumatol*	6	MMW	25	58.8	80	28.7
			HMW	18	60.4	78	29.8

FU, follow-up; BMI, body-mass-index; HMW, high molecular weight, MMW, medium molecular weight; PRP, platelet-rich plasma; LMW, low molecular weight; CCs, corticosteroids.

### Baseline of the groups

At baseline, comparability was attested (Table 2).

**Table 2:** Baseline comparability

Group	Patients (*n*)	Mean	SD	SE	Lower CI	Upper CI
VAS						
Control	230	7.6	0.1	0.0	7.6	7.6
HMW	350	6.7	1.7	0.1	6.5	6.9
LMW	42	5.8	1.2	0.2	5.4	6.2
MMW	122	7.4	0.8	0.1	7.3	7.5
Placebo	238	6	1	0.1	5.9	6.1
WOMAC						
Control	230	52.3	15.5	1.0	50.3	54.3
HMW	350	60.7	5.0	0.3	60.2	61.2
LMW	42	49.9	16.4	2.5	44.8	55.0
MMW	122	52.7	15.9	1.4	49.9	55.6
Placebo	238	50.6	11.7	0.8	49.1	52.1

SD, standard deviation; SE, standard error; CI, confidence interval; HMW, high molecular weight; LMW, low molecular weight; MMW, medium molecular weight; VAS, visual analogue scale; WOMAC, Western Ontario and McMaster Universities Osteoarthritis

### Efficacy of intra-articular HA injections

**Fig. 3:**
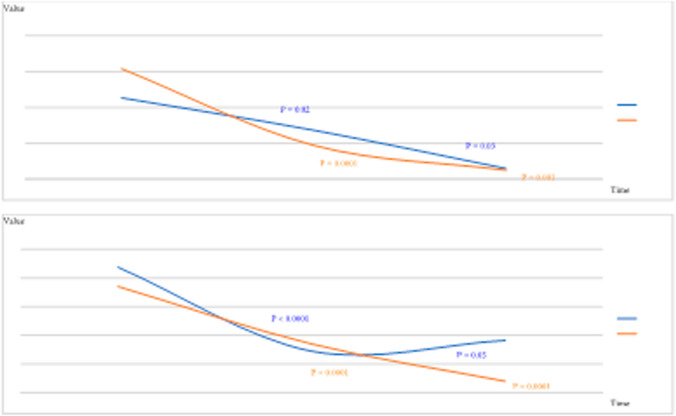
Trend of the PROMs and related *P* values at baseline and follow-ups. VAS, visual analogue scale; WOMAC, Western Ontario and McMaster Universities Osteoarthritis; MMW, medium molecular weight; HMW, high molecular weight

HMW and LMW groups statistically significantly improved in WOMAC and decreased VAS from baseline to each follow-up (*P* < 0.05). The trend of the PROMs and related P-values is shown in Fig. 3.

### Groups comparison

No between-group difference was found in VAS and WOMAC at three to 4 months of follow-up (Table 3). At 4–6 months of follow-up, the HMW group reported lower VAS compared to the MMW (*P* < 0.0001), placebo (*P* < 0.0001), and control (*P* < 0.0001) groups. The MMW group performed similarly to the placebo and control groups in VAS. Concerning the WOMAC, no difference was found between HMW and MMW (*P* = 1) at four to 6 months of follow-up, but both groups performed better than the control group (*P* < 0.0001).

**Table 3:** Between groups comparison

Groups	Means	MD	Lower CI	Upper CI	*P* value
VAS 3–4 months					
HMW versus MMW	4.7–4.5	− 0.2	− 0.8	0.4	0.7
HMW versus Placebo	4.7–4.5	− 0.2	− 0.7	0.3	0.6
MMW versus Placebo	4.5–4.5	0.0	− 0.6	0.6	1.0
WOMAC 3–4 months					
HMW versus MMW	39.4–43.6	4.2	− 1.8	10.2	0.2
VAS 4–6 months					
Control versus HMW	4.7–3.4	− 1.3	− 1.8	− 0.8	< 0.0001
Control versus MMW	4.7–4.8	0.1	− 0.6	0.8	1.0
Control versus Placebo	4.7–5.0	0.3	− 0.3	0.9	0.6
HMW versus MMW	3.4− 4.8	1.4	0.7	2.1	< 0.0001
HMW versus Placebo	3.4− 5.0	1.6	1.1	2.1	< 0.0001
MMW versus Placebo	4.8− 5.0	0.2	− 0.5	0.9	0.9
WOMAC 4–6 months					
Control versus HMW	21.5–32.5	11.0	7.5	14.5	< 0.0001
Control versus MMW	21.5–33.0	11.5	6.9	16.1	< 0.0001
HMW versus MMW	32.5–33.0	0.5	− 3.9	4.9	1.0

MD, mean difference; CI, confidence interval; HMW, high molecular weight; MMW, medium molecular weight; VAS, visual analogue scale; WOMAC, Western Ontario and McMaster Universities Osteoarthritis

## Discussion

According to the main findings of the present systematic review, the current level I of evidence suggests that intra-articular injections of HA are effective in reducing symptoms of hip OA. Moreover, HMW-HA performs better than MMW-HA, placebo and control groups at a mean of 4–6 months of follow-up.

HA is a viscoelastic molecule that exerts several positive effects in synovial fluid [67, 68]. It provides lubrication and shock absorption, demonstrates anti-inflammatory effects, has chondroprotective properties, promotes proteoglycan synthesis and scaffolding, and safeguards the subchondral area [32, 69]. The OA process induces depolymerisation of endogenous HA, reducing the distribution and concentration of molecular weight [70, 71]. The exogenous HA must ensure a substantial viscosity to sustain a thin film, facilitating the separation of cartilage surfaces while allowing them to move smoothly against each other [72, 73]. The polymer solution regions are characterised by molecular structures dependent on MW distribution, concentration, interaction between polymer chains, and solvent-polymer interactions [71, 73]. The MW of HA plays a significant role in shaping its biological properties [74, 75]. HA with a MW ranging from 2000 kDa to 4000 kDa inhibits the production of interleukin-six-induced (IL-6) matrix metalloproteinases in human chondrocytes [76]. HA with a MW below 5 kDa prompted macrophage phenotypic alterations, fostering a pro-inflammatory response [35]. Conversely, HA with a MW exceeding 800 kDa intensified changes, leading to a pro-resolving response [35].

The effects of a single intra-articular HA injection in relieving the symptoms of hip OA were analysed in a multicentric RCT with 85 patients randomly allocated to either HA or placebo [64]. No difference in VAS and WOMAC scores was observed between the two groups after 1–3 months of follow-up [64]. These results were confirmed by Brander et al. [60]. Three hundred and fifty-seven patients were randomly divided into the HMW HA group and placebo group [60]. No difference in functional parameters was observed between the two groups after 26 weeks of follow-up [60]. The first study comparing the effects of MMW and HMW HA intra-articular injection in patients with hip OA was conducted by Tikiz et al. [66]. Twenty-five patients received MMW HA injection, and 18 received HMW HA injection [66]. A statistically significant improvement in VAS and WOMAC was observed in both groups compared to the baseline after 1, 3 and 6 months [66]. No difference was observed between MMW and HMW HA injection [66]. De Lucia et al. [77] conducted a retrospective study that confirmed these findings. There was no difference in VAS and WOMAC scores between HMW and MMW over two years [77].

Given its ability to modulate the inflammatory pathway, intra-articular CCs injections have been used to manage hip OA [78, 79]. Spitzer et al. [65] compared the efficacy of intra-articular injection of HA and CCs. One hundred and fifty-six patients received a double dose of HMW HA, and 156 patients received a single dose of methylprednisolone [65]. No difference between the two groups was observed in WOMAC scores after 26 weeks of follow-up [65]. Spitzer et al. [65], focussing on a subgroup of patients with Kellgren-Lawrence III, revealed a statistically significant enhancement in functional scores among those who received HA.

The intra-articular injection of PRP demonstrated superior efficacy compared to both HA and placebo in reducing pain in patients with knee OA [45, 80]. Doria et al. [61] examined PRP and HA effects in individuals with hip OA. The study did not reveal any statistically significant difference in VAS and WOMAC scores between the two treatment groups, indicating comparable outcomes in pain and functional assessments [61].

The results of the present systematic review are confirmed by another meta-analysis conducted by Wu et al. [28] comparing LMW, MMW and HMW HA intra-articular injection in the hip. No statistically significant difference in VAS score was found between the three groups after 1 and 3 months of follow-up [28]. After a 6-month follow-up, the HMW group exhibited a statistically significant VAS score decrement compared to the MMW and LMW groups [28]. The outcomes cannot be solely attributed to the mechanical properties of exogenous HA, as its half-life within the joint ranges from a few hours to 1 month [81]. Exogenous HA can influence the rheological properties of synovial fluid, stimulating endogenous HA production and enhancing extracellular matrix protein synthesis [73, 82]. Furthermore, it possesses biological properties, modulating gene expression and exerting immunomodulatory effects [83, 84]. The MW of HA could play a crucial role in these processes, though further studies are required to determine its real influence accurately. The impact of HA extends beyond its mechanical contributions, emphasising its dynamic role in influencing both synovial fluid rheology and the intricate biological processes crucial for maintaining joint health [85].

Eymard et al. [86] analysed the influence of OA severity on the efficacy of HMW cross-linked HA injections in 97 patients with hip OA, with a 12-week follow-up. OA severity strongly influenced treatment response, particularly joint space narrowing and Kellgren–Lawrence grade. Patients with mild-to-moderate OA, lower joint space narrowing, and Kellgren–Lawrence I–II showed better pain reduction, while those with severe joint space narrowing and Kellgren–Lawrence III–IV had poorer outcomes [86]. The results of this systematic review support the role of HMW HA as a primary treatment option for managing hip OA before considering surgical intervention. Given its ease of administration, favourable safety profile, and significant symptomatic relief, intra-articular HA represents a valuable non-surgical strategy for patients with hip OA, particularly those seeking to delay or avoid joint replacement. However, its efficacy is primarily symptomatic, as it does not modify disease progression. The choice to proceed with HA treatment should be tailored to the individual patient, considering OA severity, functional demand, and concomitant joint conditions.

The present study exhibits several strengths, notably being grounded in Level I RCTs, which provide a robust and high-quality evidence base. The evaluation of the risk of bias ensures a good quality of the methodological assessment. Limited investigations on this subject are available in the current literature, lacking updates and not exclusively including RCTs. The comparative analysis with other non-operative treatments could also aid surgeons in making informed decisions.

Despite the strengths of the present systematic review, several limitations warrant consideration. Firstly, the study did not stratify results based on the degree of OA, potentially overlooking variations in treatment effects across different stages of the condition. Additionally, the relatively short follow-up period may limit the evaluation of the long-term efficacy of HA injections. Most included studies reported results within 6 months, which may not be sufficient to assess sustained benefits or the need for repeated injections. Furthermore, insufficient data on low molecular weight HA in the current literature prevented its inclusion in this systematic review, potentially leaving gaps in understanding its effectiveness. Similarly, depending on the type of treatment and HA composition, two main categories can be distinguished: linear HA and cross-linked HA. The cross-linking process allows linear HA molecules to be linked, forming structures with a higher molecular weight and reaching values comparable to those in healthy joints. However, due to the lack of quantitative data, it was not possible to analyse the potential differences in the efficacy of cross-linked HA. Another significant limitation is the heterogeneity in study protocols, including differences in the number of injections administered, injection techniques such as ultrasound or fluoroscopic guidance, and control groups, which may have influenced the comparability of results and limited their generalizability. Standardised treatment protocols would help refine clinical recommendations. A key limitation is the lack of consideration for functional profiles, including activity level and functional demand, which may significantly impact treatment outcomes. Similarly, pre-existing conditions such as femoroacetabular impingement, labral tears, and cartilage damage were not consistently reported across studies, making it difficult to determine their influence on the effectiveness of HA injections. Potential publication bias and selective outcome reporting must also be considered. The risk of bias assessment indicated an overall low-to-moderate risk, but some studies raised concerns regarding blinding and outcome measurement. Future studies should include longer follow-ups to determine the persistence of clinical benefits and the need for repeated injections, as well as standardised protocols to reduce heterogeneity and improve the comparability of findings. Further research should better assess the role of activity level, functional demand, and pre-existing conditions to refine patient selection and optimise treatment strategies. Finally, efforts should focus not only on symptomatic treatment but also on therapies targeting the underlying mechanisms of OA, aiming to slow disease progression and modify its natural course. High-quality clinical studies investigating disease-modifying approaches are needed to advance the management of OA beyond symptom relief.

## Conclusion

Intra-articular injections of HA are effective in reducing symptoms of hip OA. Moreover, high molecular weight (HMW) HA performs better than medium molecular weight (MMW) HA, placebo and control groups at a mean of 4–6 months of follow-up.

## Supplementary Information

Below is the link to the electronic supplementary material.

Supplementary file1 (DOCX 18 KB)

## Data Availability

The datasets generated during and/or analysed during the current study are available throughout the manuscript.
