# Supplementary material for: Intra-articular hyaluronic acid injections for hip osteoarthritis: a level I systematic review
Source: Eur J Orthop Surg Traumatol. 2025 May 9;35(1):180. doi: 10.1007/s00590-025-04292-7 (PMC12064613; doi:10.1007/s00590-025-04292-7)
Supplement: Supplementary file 1 — Supplementary file1 (DOCX 18 KB) [file 590_2025_4292_MOESM1_ESM.docx]

**Systematic literature search for:**

**Efficacy of intra-articular hyaluronic acid infiltrations for hip osteoarthritis: a level I meta-analysis**

**Concept 1: Hip osteoarthritis**

**Keywords:**

Hip OA

Hip osteoarthritis

**Mesh:**

"Osteoarthritis, Hip"[Mesh]

**Concept 2: Treatment**

**Keywords:**

hyaluronic acid infiltrations

HA infiltrations

Low molecular weight (LMW)

medium molecular weight (MMW)

High molecular weight (HMW)

**Mesh:**

"Hyaluronic Acid"[Mesh]

**Concept 3: Interest of the outcome**

**PROMs**

**Keywords:**

Western Ontario and McMaster Universities Osteoarthritis (WOMAC)

Visual analogue scale (VAS)

**Mesh:**

"Patient Outcome Assessment"[Mesh]

"Patient Reported Outcome Measures"[Mesh]

**Concept 1:**

"Osteoarthritis, Hip"[Mesh] OR hip osteoarthritis OR hip OA

**AND**

**Concept 2:**

"Hyaluronic Acid"[Mesh] OR hyaluronic acid infiltrations OR HA infiltrations OR low molecular weight hyaluronic acid OR LMW OR medium molecular weight hyaluronic acid OR MMW OR high molecular weight hyaluronic acid OR HMW.

**AND**

**Concept 3:**

"Patient Outcome Assessment"[Mesh] OR "Patient Reported Outcome Measures"[Mesh] OR PROM OR visual analogue scale OR VAS OR Western Ontario and McMaster Universities Osteoarthritis OR WOMAC

**NOT**

knee

**Summary of the Search**

((("Osteoarthritis, Hip"[Mesh] OR hip osteoarthritis OR hip OA) AND ("Hyaluronic Acid"[Mesh] OR hyaluronic acid infiltrations OR HA infiltrations OR low molecular weight hyaluronic acid OR LMW OR medium molecular weight hyaluronic acid OR MMW OR high molecular weight hyaluronic acid OR HMW.)) AND ("Patient Outcome Assessment"[Mesh] OR "Patient Reported Outcome Measures"[Mesh] OR PROM OR visual analogue scale OR VAS OR Western Ontario and McMaster Universities Osteoarthritis OR WOMAC)) NOT (knee)
